# The Enactment of Care: Lessons Learned From a Multisector Coalition Advocating for Those Living in Senior Housing

**DOI:** 10.1093/geroni/igab046.1458

**Published:** 2021-12-17

**Authors:** Tam Perry, Claudia Sanford, Dennis Archambault, Michele Waktins, Zach Kilgore, Michael Appel

**Affiliations:** 1 Wayne State University, Detroit, Michigan, United States; 2 United Community Housing Coalition, Detroit, Michigan, United States; 3 Authority Health, Detroit, Michigan, United States; 4 Volunteers of America-Michigan, Southfield, Michigan, United States; 5 CSI Support & Development, Warren, Michigan, United States; 6 Develop Detroit, Detroit, Michigan, United States

## Abstract

This presentation explores how a coalition, Senior Housing Preservation-Detroit, considered and planned for “care” in senior buildings in Detroit, Michigan. Detroit was one of the American cities affected in the early days of the pandemic; the coalition pivoted its work in creative, collaborative ways which included understanding the rapidly changing context for those living in low-income senior buildings. Older minority adults have been shown to be disproportionally affected by COVID-19; the coalition successfully advocated for testing to be brought to senior buildings (and now vaccine distribution) and addressed mask distribution and food insecurity in several senior buildings (see Archambault, Sanford and Perry, 2020). Without the long-established partnerships, “care” could not have been as coordinated, multi-sector and trusted. The presentation will discuss lessons learned that can be applied to future challenges in supporting the well-being of residents as they negotiate their residential spaces.

